# Qualitative Process Evaluation of a School‐Based Group Intervention (DISCOVER) for Depression and Anxiety for Older Adolescents

**DOI:** 10.1002/jad.70083

**Published:** 2025-11-28

**Authors:** Tim Weaver, Sorcha Alford, Helen Gleeson, Irene Sclare, Stephen Lisk, June S. L. Brown

**Affiliations:** ^1^ Middlesex University London UK; ^2^ King's College London London UK; ^3^ South London & Maudsley NHS Trust London UK; ^4^ Kings College London London UK

**Keywords:** accessible, cognitive behavioural therapy, evidence‐based intervention, older adolescents, process evaluation, qualitative research

## Abstract

**Introduction:**

DISCOVER is a Cognitive Behavioural Therapy based intervention for anxiety and depression in 16‐18‐year‐olds delivered in schools, shown to be clinically and cost‐effective by the Brief Educational workshops in Secondary Schools Trial (BESST). DISCOVER comprises pre‐workshop 1:1 meeting, a group workshop, and 1:1 follow‐up phone call. A qualitative process evaluation investigated intervention delivery and outcome generation within BESST.

**Methods:**

Thematic analysis of interviews with an ethnically diverse sample of male and female students (*n* = 22) and focus groups with DISCOVER practitioners (*n* = 21) in 4 English regions.

**Results:**

Practitioners valued the pre‐workshop student meetings and workshop materials. However, they felt uncomfortable with the scripted delivery, and found delivery of all content difficult in the time available. Time constraints and variable adherence to follow‐up phone calls limited provision of goal‐attainment support. Students preferred relatable, interactive workshop elements more than didactic teaching. They found group discussions ‘normalising’, but often reported fatigue by the workshop end. Some reported beneficial use of thought challenging, mindfulness and sleep hygiene techniques, particularly during exams, but reported variable experience of follow‐up calls. The DISCOVER app was rarely accessed. Male and female students provided similar accounts.

**Conclusions:**

Students valued DISCOVER and perceived it as effective. Practitioners expressed support for implementation in routine practice. Findings suggest enhanced effectiveness may be achieved by (a) reviewing the parameters for modification of scripted material; (b) streamlined delivery of workshop content, and (c) enhancing support for goal‐attainment by providing extra practitioner training and using more digital and telecommunication resources to boost student engagement.

## 1 Introduction

Adolescence is a time of critical cognitive and neurobiological development. These developmental changes occur as young people navigate high stress environments in which the imperative to achieve social integration, academic success and transition to further education or employment figure prominently [Arnett 2000]. Anxiety and depression are increasingly prevalent in adolescent populations. The proportion of 17‐ to 19‐year‐olds in England with a probable mental disorder increased from 17.4% in 2021% to 25.7% in 2022 [Newlove‐Delgado et al. 2022]. For around 1 in 12 of this age group, these disorders are associated with high levels of distress, significant impact on education and daily life [Marcheselli et al. 2018], and an increased risk of self‐harm and suicidality [Hawton et al. 2012; Rahman et al. 2021]. Almost three‐quarters of adult mental disorders have a first onset before age of 18 [Kessler et al. 2005].

Without effective intervention that provides young people with the skills and techniques to manage these mental health problems, they may seriously impact a young person's development, increasing their future risk of poor health, impaired educational performance [Kochel et al. 2012; Patalay et al. 2015] and a range of negative social and occupational outcomes [Fergusson and Woodward 2002].

Despite this morbidity, it is acknowledged that National Health Service (NHS) mental health provision lacks the capacity and flexibility to meet the needs of young people [Department of Health 2015]. Recent estimates suggest that 60% of children and young people in the United Kingdom with a diagnosable mental health condition do not receive care through specialist Child and Adolescent Mental Health Services (CAMHS). [Department for Health and Social Care 2022.]

These data indicate a critical need for effective, accessible interventions for adolescent populations, to alleviate current distress, and reduce the risk that adult mental health problems may develop or become severe. These concerns have led to the development, in England, of Mental Health Support Teams (MHST), staffed by a new workforce of Educational Mental Health Practitioners (EMHPs) and Senior Wellbeing Practitioners (SWPs) (usually employed at NHS Band 5 and 7 respectively) who are trained to deliver evidence‐based interventions within schools [Department of Education 2023]. The first MHSTs were commissioned in 2018 and by 2022 it was estimated that 26% of pupils went to schools with a linked MHST [Department of Education 2023].

While an international review of school‐based mental health interventions reported only modest benefits, it suggested that Cognitive Behavioural Therapy (CBT) based interventions delivered by clinicians in secondary schools were most likely to be effective [Zhang et al.^2023^]. While the clinical evidence in relation to the 16+ age group is limited to one small trial [Hains and Szyjakowski 1990], delivering interventions within schools can increase accessibility and reduce stigma [Patel et al. 2007].

### 1.1 The DISCOVER Programme

Responding to the absence of evidence‐based interventions for older adolescents, the DISCOVER programme was developed to provide a within‐school, CBT‐based intervention for 16–18‐year‐old students [Sclare et al. 2014]. Delivering DISCOVER in a workshop format in schools is intended to promote accessibility to diverse groups. Its self‐referral pathway is designed to enable autonomy, promote acceptability and reduce stigma [Brown et al. 2010].

After a successful feasibility study [Brown et al. 2019], a full trial of DISCOVER was undertaken (Brief Educational Workshops in Secondary Schools Trial (BESST)). Hence, DISCOVER has progressed through conceptual development, feasibility assessment and evaluation phases in a manner consistent with current guidance on the development of complex health interventions [Skivington et al. 2021]. The BESST methodology is described elsewhere [Lisk et al. 2022] but in brief the trial employed a cluster randomised design and evaluated the clinical and cost‐effectiveness of DISCOVER compared to treatment‐as‐usual. DISCOVER was delivered by MHSTs who received 2‐days of specialist training. The primary clinical outcome was symptoms of depression assessed at 6‐month follow‐up.

The central component of DISCOVER is a 1‐day group workshop delivered on the school premises. The workshop focuses on personal and academic stresses and offers CBT techniques for managing anxiety and mood problems. Interactive discussions aimed at normalising young people's experiences are complemented by video, games, take‐home materials and a web‐based app all of which aim to enhance accessibility of the material to the age‐group.

DISCOVER was designed to be congruent with the increasing development of personal autonomy and self‐determination that characterises the actions, motivations and preferences of adolescents [Pao 2017]. Its self‐referral pathway, without parental consent, is designed to promote autonomy and empowerment, increase acceptability and reduce stigma [Brown et al. 2010]. DISCOVER also features personal goal planning, setting and review as a key component. Before the workshop, MHST practitioners meet self‐referring students individually and discuss goal planning. This is re‐visited briefly in an individual reflective task at the start of the workshop. Goal setting is done at the end of the workshop, when the student is helped to set achievable personal goals and identify CBT techniques taught in the workshop which support this. Practitioners then offer follow‐up phone‐calls to monitor student's progress, review personal goals and support students to apply the chosen techniques [Michelson et al. 2016].

BESST included a qualitative process evaluation which is reported in this paper. Trial investigators followed Medical Research Council (MRC) guidelines on the evaluation of complex interventions [Craig et al. 2008; Skivington et al. 2021] and embedded a qualitative process evaluation within the study design [Moore et al. 2015].

The aims of the process evaluation were to provide a contextualised analysis of the process of intervention delivery and outcome generation within the BESST trial. The specific objectives were:

a. To investigate the experience of students who received DISCOVER, and the teams that delivered DISCOVER;
b. To identify contextual and process factors that support or hinder delivery of the intervention with fidelity,
c. To identify mechanisms of action that influence the generation of outcomes observed by the trial, and,
d. Assess the potential for translation of DISCOVER to routine practice.

The trial findings showed that DISCOVER was a clinically and cost‐effective intervention that reduced the symptoms of depression, particularly amongst those who exhibited elevated symptoms at baseline. DISCOVER was also found to reduce anxiety, improve wellbeing and resilience. [Brown et al. 2024; Lisk et al. 2024]. The investigators concluded that DISCOVER showed promise as an early intervention, and that further research should assess its potential for wider implementation [Brown et al. 2024].

## 2 Methods

### 2.1 Study Design

Following MRC guidance our process evaluation focused on investigating the implementation and delivery of the intervention, identifying potential mechanisms of action and the influence of contextual factors [Moore et al. 2015]. The process evaluation employed a qualitative methodology. We conducted semi‐structured interviews with students who received the DISCOVER intervention, and interviews or focus groups with the practitioners (mostly EMHPs) from the MHSTs which delivered DISCOVER.

### 2.2 Participants and Recruitment

#### 2.2.1 Practitioners

Each of 15 MHSTs trained to deliver DISCOVER in the BESST trial was invited to take part in a focus group. The ‘Delivery Teams’ comprised 2–3 EMHPs and 1 or more SWP who provided management and supervision to the EMHPs. When an MHST agreed to participate, we sent each DISCOVER‐trained practitioner in the team an information sheet and obtained their signed consent before data collection. The intention was to conduct a focus group (or joint interview) with members of each delivery team, but we took a pragmatic approach to data collection and where a small team‐based focus group was not possible, we interviewed two staff jointly or a single team member individually.

#### 2.2.2 Students

The clinical team which developed DISCOVER has offered the workshops in Further Education (FE) colleges. However, the breadth and timetabling of courses in FE colleges has made it difficult to offer day‐long workshops to a sufficiently large number of students who have variable patterns of attendance and may also include adult learners. For these reasons the trial was conducted in schools only.

Students were recruited from 8 schools which were randomised to the intervention arm of BESST and received DISCOVER during the first year of the trial (the academic year 2021–22). To avoid scheduling clashes with the BESST 6‐month follow‐up interviews (i.e. the trial primary outcome assessment) and the students' end of year examinations, qualitative interviews were scheduled for late June/early July 2022. (Earlier interviews were not possible because we needed the trial Research Assistants to approach students, which unblinded them to group allocation. For this reason, interviews had to be undertaken after trial assessments were complete, when unblinding was no longer a concern.) Although managing exam stress was not a key aim of DISCOVER, this timing provided students an opportunity to reflect on the utility of the intervention during the high‐stress period around the exams. The disadvantage was that students were not always available to be interviewed outside of term time, and we had to employ an opportunistic sampling method.

From each school, we invited 8 randomly identified students representing both genders, to contact us if they would be willing to take part in an interview. We aimed to interview 2 students from each school (i.e. a minimum sample of 16 students, ideally 1 male and 1 female.) However, in five school schools we increased the sample size in an attempt to recruit male students who did not initially come forward.

### 2.3 Data Collection Method

**Figure 1:**
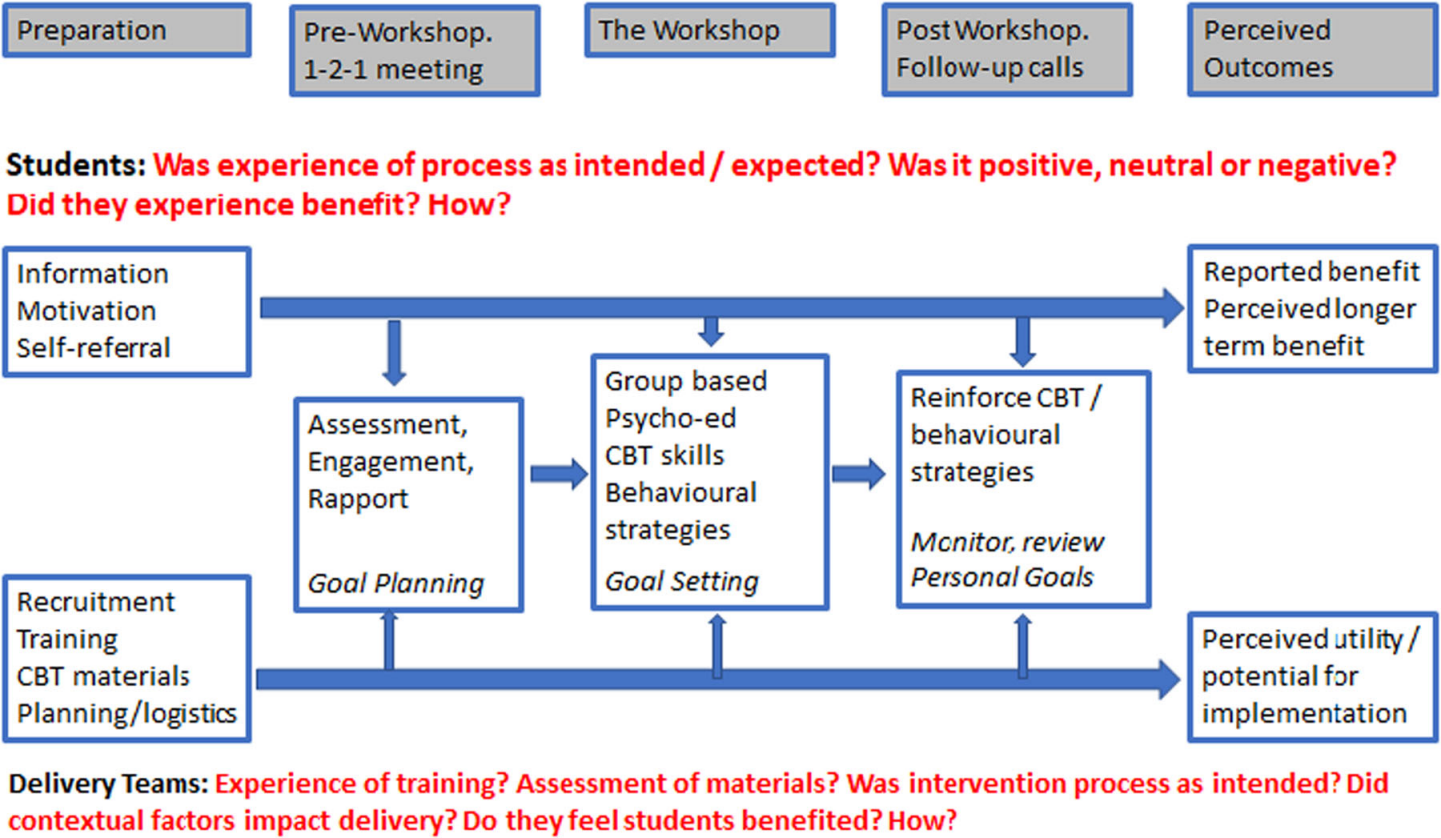
Thematic framework for qualitative data analysis.

All interviews and focus groups were semi‐structured employing topic guides that were applied with flexibility to ensure coverage of key themes, while providing a framework within which emergent themes could be explored. Both student and practitioner topic guides were designed firstly, to explore the structured, three‐stage intervention process (See Figure 1.), and secondly, to assess the experience of participants exposed to the intervention.

Students were asked about their experience of the intervention process, whether or not they felt benefit from DISCOVER, in what ways, and whether any specific materials, components or characteristics of the intervention were perceived to have been critical. (e.g., issue‐specific content such as that focusing on sleep, different learning modalities such as group discussion or videos, experience of the group format and interaction with peers.) Questions were posed neutrally to illicit accounts of their experience of the intervention and the perceived utility, of each stage of the intervention. The primary focus was upon whether students experienced the benefits anticipated in terms of mental health (i.e. the primary and secondary clinical outcomes) and whether they acquired the skills and techniques intended to help achieve and sustain improved mental health (i.e. the mechanisms of action). Questions also included exploration of what was ‘liked’ and ‘helpful’ or ‘disliked’ and ‘not helpful’ about the intervention. Interviewers explored both positive and negative accounts to identify any emergent themes. In this way discussion of potentially adverse effects was facilitated and (if identified) could be explored by further questions and probing.

Reflecting the focus on implementation and process, practitioners were asked to assess the DISCOVER training they received, the overall quality of the materials provided, their experience of the intervention process, the perceived benefit to students, and the potential for DISCOVER to be implemented in routine practice.

Student interviews were completed by a research worker between June and August 2022 and lasted between 30 and 50 min. Practitioner focus groups and interviews lasted up to 60 min and were completed online by TW between February and April 2023. All interviews and focus groups were completed online via Zoom or Teams.

### 2.4 Data Management and Analysis

Interviews and focus groups were audio recorded and professionally transcribed verbatim. Transcripts were analysed using Thematic Analysis [Braun and Clarke 2019; Braun and Clarke 2022]. This involved reading the transcripts as a whole to achieve overall familiarisation with the data before moving on to formal data coding. Within this, both inductive and deductive approaches were followed in the generation of themes. Given that the imperative of the process evaluation was to elaborate and contextualise the intervention process and the process of outcome generation within the trial, some clear a priori questions shaped the thematic framework. Notably, the DISCOVER intervention has three distinct phases—the 1:1 pre‐workshop meeting, the group‐based workshop (with its various component parts) and the 1:1 phone follow‐up. This dictated a structured approach to first‐level coding which mapped to this process (See Figure 1.)

Working under the supervision of TW, SA and HG respectively analysed the student and delivery team data. Data that related to research question about how the two participant groups experienced each stage were coded following an inductive approach to closely represent participants' experiences of delivering or receiving DISCOVER. This also facilitated data triangulation from the different perspective of provider and recipient.

Once the data were organised according to these primary ‘process’ codes, secondary themes were generated through a reflective analysis supported by discussion between the co‐authors. Reflective processes were an integral part of the analysis, making sure that discussions took place before finalising coding decisions throughout the analytic process. The resulting themes were reviewed, defined and finalised when they were considered a coherent and authentic reflection of participants’ experiences.

### 2.5 Ethical Review

The study was reviewed and approved by King's College London Psychiatry, Nursing, and Midwifery Research Ethics Subcommittee (HR‐20/21–17758).

## 3 Results

### 3.1 Sample Characteristics

Across the 15 MHSTs, a total of 69 practitioners were trained to deliver DISCOVER. Eleven teams (73%) responded to the invitation to participate in the process evaluation. When recruitment was undertaken, 37 DISCOVER trained staff worked at these 11 teams of whom 21 (57%) participated in either an interview or focus group. Practitioners from 2 teams took part in (small) focus groups comprising three colleagues. Joint interviews were completed with two practitioners from 6 teams, while the remaining 3 teams were represented by 1 team member who completed a 1:1 interview.

Three SWPs participated (1 in each of the two small focus groups, and 1 in a joint interview.) All other participants were EPMHs. All but 1 of the participants were female, the exception was a male SWP. The participating practitioners had delivered DISCOVER 1–3 times (median = 1).

A sample of 22 students were interviewed (*n* = 6 male, *n* = 16 female). At least 2 students were recruited from each school (range 2‐4). We wanted to select 1 male and 1 female from each school. At three schools, 1 male and 1 female student came forward and were successfully interviewed. At five schools, 2 female students initially came forward (and were interviewed). At these schools, further efforts were made to recruit males. At one school, we successfully recruited a third student who was male, while at another school, 2 males came forward simultaneously and were both interviewed. At the three remaining schools, no males came forward. However, one additional female came forward at each school, and it was decided that we had the capacity to include these 3 additional female students in the sample. Hence, our achieved sample was 22, comprising 6 males from 5 schools and 16 females from 8 schools.

### 3.2 Thematic Framework

DISCOVER has a clear structure and sequential process. Within BESST this involved (a) the pre‐workshop 1:1 meeting (b) the 1‐day group‐based workshop and (c) a 1:1 follow‐up phone call to each student. Each of these stages in the intervention process represented a key process theme for both practitioners and students. Accordingly, in presenting the results we integrate the data obtained from both student and practitioner perspectives under these process themes.

In addition, for both participant samples ‘preparation’ emerged as a key theme. Practitioners ‘preparation’ focused on training and on their assessment of the intervention materials. For student's ‘preparation’ concerned their motivation to participate, the information they received before participation and their attitudes towards the intervention format.

The final key theme for both students and practitioners was ‘perceived outcome’. Both groups included reflections on the perceived benefit of DISCOVER and its potential for wider implementation. This thematic framework is summarised in Figure 1 below.

## 4 Theme 1: Preparation

### 4.1 Practitioner Experience

#### 4.1.1 Training

Training for the workshops spanned 2 days, was perceived to be well organised and delivery teams felt well prepared. Most teams reported that 2 days of training was difficult to accommodate within already busy workloads, though for some this view was coloured by the small number of workshops that were delivered relative to the time invested in training.

#### 4.1.2 Workshop Materials

Many noted that the delivery style, activities and videos, were appropriate to the target age group and that the presentation of the material was novel and well designed.

The content is quite familiar … but it was just a different way of putting that together that was quite engaging to the young people.

(EMHP, Team 2)

However, a large proportion expressed reservations about the scripted nature of the workshop materials. The most common concern was that this would restrict their ability to draw on previous experience and express their own delivery style. Some felt this was apparent when they did then deliver the workshop.

… it added a bit of an anxiety … thinking ‘oh gosh I've got to keep on top of this.’ And some bits just felt very unnatural to the way I might deliver it … I felt like it created a bit of barrier as well because I was then reverting to script and not feeling I was fully sort of with the room.

(EMHP, Team 06)

However, some felt the script helped reduce the pressure of the additional workload and was seen as helpful when delivering a new intervention with limited preparation time. Whatever their personal view, most practitioners understood the rationale for the scripted materials and, mindful of the imperative to deliver the trial intervention with fidelity, they adhered to it.

### 4.2 Student Experience

#### 4.2.1 Perceived Need

Most participant self‐reported some mental health issues and described experiences of “*depression*”, “*anxiety*”, “*stress”*, “*breakdowns*” or “*panic attacks*” which sometimes significantly impacted their lives and education.

It affected everything, really. I was never motivated to do anything. I didn't properly take care of myself. I didn't sleep properly. I didn't do proper hygiene … I was not even doing homework.

(Student 10)

All students identified a specific personal need for support. This ranged between managing stress and anxiety in relation to deadlines and exams, through to wanting help with balancing personal and school life, sleep issues and procrastination.

I've barely ever revised, which has come to my detriment in some exams. So, I'd say that that's, kind of, like, a big problem.

(Student 5)

Most students were aware that they should try to manage their poor mental health. They also anticipated bouts of declining mental health (e.g., in exam periods) and wanted to address this. Hence, students recognised their need for help and support, and the probability that without this their mental health might deteriorate further as they encountered stressful, high‐pressure life events.

#### 4.2.2 Motivation for Participation

Students generally presented with good mental health literacy. They were sometimes aware of (and referred to) interventions such as CBT. But despite awareness of their mental health problems, many students reported not having the ‘tools’ to address these issues or were unaware of how to access support. A small number of students expressed a need for greater support than they were currently receiving.

Most students expected to learn new and helpful ‘tools’ in the workshop, though others (including some reporting previous exposure to mental health interventions) were more sceptical. Regardless of prior experience most students approached DISCOVER with an “open mind”. Even though some of these students had low expectations.

I thought, ‘Well, if it works, it works, and if it doesn't, it doesn't’.

(Student 5)

While this need to address personal mental health issues was common, several students identified a more altruistic motivation, wishing to be involved in research with potential to make a positive difference. There were also reports of the positive influence of peer groups, with some describing a culture of mental health literacy and motivation to seek help.

## 5 Theme 2: Pre‐Workshop Meeting

### 5.1 Practitioner Experience

There were mixed experiences of the pre‐workshop meeting. Some teams felt that the planned 30‐min meetings with each student were rushed and they had too many students to speak to. Most agreed however that it gave the delivery team a good sense of the common issues experienced by students which enhanced workshop delivery. However, some practitioners reported having limited time to focus on goal planning.

Commenting on the self‐referral pathway, practitioners felt that most students who participated were appropriate and had mild mental health concerns. However, a small number of students who required more support were identified at pre‐workshop meetings. Teams described particular attention being given to these students, observing their presentation at the workshop and providing extra postworkshop follow‐up calls.

…it was definitely essential to have that (pre‐workshop meeting) and it was just about the right amount of time as well that we spent with them and we got to know them a little bit. And there were some things that were quite important to know before they came to the session.

(EMHP, Team 06)

### 5.2 Student Experience

Students generally provided little commentary on the pre‐workshop meeting. When asked about meetings before the workshop, for most students, the significant interactions were the school assembly (when information about the trial was first provided) and their interview with a trial researcher. The pre‐workshop meeting was generally seen as a positive opportunity to meet with the workshop leader which some felt made interaction at the workshop easier. Almost universally students felt very well briefed by the verbal and written information they received. However, very few reported any form of explicit ‘goal planning’ at their session.

## 6 Theme 3: The Workshop

### 6.1 Practitioners

Views on the workshop activities were positive. Many mentioned the quality and relatability of the videos and also noted good student engagement with sections addressing ‘thoughts, feelings and behaviours’, sleep hygiene and procrastination.

Workshop elements where the delivery teams felt engagement was less successful were those involving delivery of scripted, educational content. Some reflected on their own lack of engagement while pre‐occupied with ‘following the script’ rather than interacting with the students.

I remember us all being, like, trying to follow the script because we knew that's what we needed to be doing, but then we also didn't want to be there reading through the script because we wanted to be engaging for the young people.

(EMHP, Team 2)

All delivery teams reported challenges in covering the volume of workshop material in the allotted time, and many felt the absence of time for discussion and reflection restricted full engagement from students.

… it was a lot to cover in 1 day … there wasn't enough time.

(EMHP, Team 11)

This also meant some delivery teams did not have time to adequately support goal setting at the end of the workshop day.

… goal setting was massively compromised by it … it was very quick.

(EMHP, Team 7)

However, those who delivered a second or third workshop, reported that as they became more familiar with the content, subsequent workshops felt less rushed and more relaxed. Some attributed this to knowing the structure of the sessions better. For some this allowed them to add their own questions and comments which made the delivery more ‘natural’ for them.

That's what I really struggled with ‐ just not being able to show your personality because I think that's how you get these children to speak and ultimately, help them … we did have to go off script as such in the second one and it was better.

(SWP, Team 01)

The gender mix of groups varied across schools, no notable differences in levels of engagement between genders was reported by any of the delivery teams.

### 6.2 Student Experience

#### 6.2.1 Group Format

The workshop group format received mixed reviews from students. The overall environment of the workshops was described as “friendly”, “warm”, “confidential”, “relaxed”, “safe” and “respectful” with most students feeling they were all there for a shared goal. Some who reported feeling initial reserve, said they ‘opened‐up’ as the day progressed. Most students felt the group provided a valued opportunity for discussions which one described as the “highlight of the workshop”. Many reported that hearing their peers discuss similar issues was normalising, deepened their understanding and provided them with new perspective and alternative coping mechanisms. For most, the fact that the other students were from the same school cohort meant that their experiences were relevant and relatable.

We're all dealing with similar stress situations and then realising that actually, like many people around me, have similar problems regarding stress and that everyone's also looking for ways to improve and to find ways to solve or to manage stress. It was really nice.

(Student 1)

However, a minority were concerned about the lack of confidentiality and anonymity within a group of students who knew one another. Two students reported fearing judgement or stigma from other attendees some of whom they had previous conflict with.

The gender mix of each workshop was valued by all. Male and female students provided very similar accounts of their experiences in the workshops. Some female students praised the males who were present for being open and reflective—sometimes contradicting their expectations. While some students preferred to be passive and largely silent participants, no students reported any significant problems (such as domineering or disruptive behaviours) which impacted their ability to engage to a level they felt comfortable with.

#### 6.2.2 Workshop Content and the Delivery Team

Several students reported that the delivery of DISCOVER across 1 day was ‘too much’, and reported feeling tired and unable to concentrate by the end. Some students suggested splitting it across sessions. Echoing the concerns of some practitioners, two students reported that some parts of the workshop delivery felt ‘robotic’ and ‘scripted’.

The workshops provided a combination of videos, interactive activities and workbook tasks. These were widely viewed as positive, and prevented the workshop from being too ‘lecture‐like’. They appreciated that students could tailor what they took from the workshop to meet their personal needs.

Because they offered such a wide range of methods to help with stress and exams, I think it reached to a lot of people… I think everyone at least took one away.

(Student 19)

One student described the videos as ‘cringey’ and ‘a bit cliched’ (Student 20), and another felt the workshop did not address their needs around race and sexual identity (Student 6). However, these views did not reflect the majority response, which was largely positive. Many of the students related to the characters in the videos, and some felt they normalised their issues and made them feel less alone in their struggles.

It was nice to see that I wasn't alone in a way. Like even though they were actors, these are things that happen to people and these are the things that people go through. So, it was nice to see that inclusivity.

(Student 7)

The delivery team were described as “kind”, “friendly”, “lovely” and “funny”. Students reported an appreciation that external staff delivered the workshop because, unlike school staff they did not know them, and would not see them postworkshop. This anonymity seemed to be valued by students and encouraged a degree of openness and candour.

## 7 Theme 4: Follow‐Up Calls

### 7.1 Practitioners Experience

Most of the delivery teams reported challenges in undertaking follow up calls. Most felt the time had not been factored into their workloads before delivering the workshops. They often found several attempts were needed to reach some students and found it time consuming to make repeated calls. Some reported that they made one attempt to call, but if they could not make contact they did not persist.

The perceived utility of the calls seemed to vary by team and school. Several practitioners reported how students had told them about achieving their goals, using learned techniques to reduce anxiety, improve sleep and exam revision. However, others stated that many students were unable to remember the goals they had set, or whether they had yet to try to use the techniques learnt on the day. Some considered that a majority of students may not have wanted or needed the calls.

… (students) just didn't feel the need to have these check‐in follow‐up calls afterwards because they'd already done the workshop and I think lots of them didn't really understand why they were being called.

(EMHP, Team 2)

Despite reservations about workload and the utility of the calls, most practitioners would have preferred an ‘in‐person’ follow up with those students who wanted or needed the follow‐up. No respondents found that students were engaging with the DISCOVER app at follow‐up.

### 7.2 Student Experience

Not all students reported receiving a follow‐up call. Of those who did, most valued the opportunity to discuss their issues with confidentiality and assess progress with personal goals.

(the calls) were my favourite part of the whole thing. Because each one of them left me with something to do. So, we wrote those goals down, and they were to see how we were getting along with them.

(Student 14)

Some students reported not receiving a call but said they would have valued the 1:1 discussion. However, others said they did not answer the call due to bad timing, apprehension about lack of privacy or uncertainty about an unknown number. Two students reported a dislike of speaking on the phone and that they would have preferred a face‐to‐face meeting.

The workbooks were well received as they gave the students the opportunity to record any thoughts or useful exercises and provided a reference point for their future use. One student reported passing the workbook on to their younger sibling. In contrast, only 1 student reported using the DISCOVER app, finding it useful to use before and after the follow‐up calls. All other students reported either disliking the format, or not bothering to download it.

## 8 Theme 5: Perceived Benefit, Future Application

### 8.1 Practitioner Perspective

Despite reservations described above, delivery teams felt that students benefitted from the workshops. Delivery teams noted that the workshops addressed issues that students were experiencing and that the techniques and activities were appropriately designed for the age group.

I didn't think when reading the manual that it would be as well received and as helpful. But I think definitely having those videos and it being relatable to the young people really helped and I think they did get a lot from it.

(EMHP, Team 9)

While some were concerned about how DISCOVER would be incorporated into their existing workloads, they were almost universally supportive of routine implementation. The most common recommendation was to build in more time for the workshops. There was general agreement that a 2‐day delivery would allow for fuller engagement of students, give time to reflect on the content and in particular help students to work on their personal goals.

### 8.2 Student Perspective

Students expressed mixed views about whether DISCOVER provided any enduring benefit, but only a small minority felt it had not been helpful. Many students were positive and enthusiastic about the impact DISCOVER had on their lives. Many felt empowered to open‐up about their mental health and for others it provided a springboard to seeking further resources and/or help.

In response to questions about intervention components that had particular value, some students reported regular use of thought challenging, mindfulness and sleep hygiene techniques. Some students reported having shifted lifestyle to achieve a healthier work‐life balance and envisaged using the skills learnt in the long term. Many of the students, including those who were less positive about the longer‐term impact of the workshop, stated that advice on prioritising, time‐management, thought challenging, timetabling and stress reduction had been very helpful during their exams. Indeed, multiple students reported successfully using mindfulness or meditation techniques when having ‘anxiety’ or ‘panic attacks’ before an exam.

… increasing anxiety happened when I didn't use them but then I started using them again and I was, like, ‘Oh, I'm calm again, I can function’.

(Student 16)

## 9 Discussion

The aim of the process evaluation reported here, was to provide a contextualised analysis of the process of intervention delivery and outcome generation within the BESST trial. To achieve this, we implemented a qualitative study which investigated the experience of students who received DISCOVER, and the MHST staff that delivered it. Our analysis identifies a number of mechanisms of action that potentially influenced the generation of the outcomes observed by the trial. This enhances our understanding of how DISCOVER operated in the trial setting, and potentially how it could be delivered in routine practice.

First, MHST staff regarded the DISCOVER training as rigorous and high quality and were well prepared to deliver the intervention.

Second, MHST staff expressed widespread misgivings about the scripted delivery of the workshop content. While they generally reported adherence to the given script, those who completed multiple workshops conceded that they began to adopt a more flexible approach to delivery of the material which was more comfortable for them and perceived to be more engaging to students. Echoing this, students described how they enjoying the discussion and nondidactic content, such as the videos, in contrast to the scripted delivery of some material.

It should be noted that the model of training employed in BESST contributed significantly to the costs of delivering the intervention [Brown et al. 2024]. Increasing the number of workshops each team delivered would clearly provide a larger return on this investment, but alternative approaches to training—either through Continuous Professional Development (CPD) or embedding the training in the EMHP or SWP curriculum would need to be considered if DISCOVER were to be delivered in routine practice. This may have a favourable impact on future health economic calculations.

Third, many MHST staff reported struggling to deliver the full workshop content in the time available, and students often reported fatigue by the end of the workshop. While spreading the workshop over 2 days (a suggestion made by some MHST staff, but not students) would superficially address this problem, it might generate others challenges. Scheduling, disruption of teaching, room availability, achieving consistent student attendance and the additional cost of MHST staff time being the most notable foreseeable problems.

While careful review of the workshop content may provide some opportunities for streamlining the overall delivery time, caution should be exercised. One of the clear messages from the student interviews was that they valued the range of the content covered and that this increased their awareness of the diverse challenges their peers experienced. This was an important element in normalising their own distress. They also valued the multiple tools and strategies the workshop taught. Different students were able to select from a menu to find what worked for them, while some described using several different strategies.

Fourth, it is possible that the time pressure discussed above had a particularly negative impact on the planning, setting and review of goals. This was a key element of DISCOVER, but it is unclear whether ‘goal planning’ was done consistently in the pre‐workshop 1:1, and it was often reported that ‘goal setting’ at the end of the workshop became compressed due to lack of time. These problems may be compounded by the relatively small proportion of students who received, or accepted, the follow‐up phone calls intended to review personal goals. Trial fidelity data indicates that overall less than 50% of the intervention groups received a follow‐up phone call [Brown et al. 2024]. Although some students saw little value in goal setting, others who made and reviewed personal goals valued this highly, and perceived it to have had a significant positive impact on their mental health. On the basis of both the trial fidelity data and the qualitative findings presented here, it is difficult to avoid the observation that goal setting may have failed to achieve its maximum potential for impact in the trial.

Finally, while the longer‐term impact of DISCOVER is unknown it is noteworthy that the student's largely positive accounts were obtained 2–3 months after the trial outcomes were assessed. Though not designed as an intervention to address exam stress, it is encouraging that a proportion of the students interviewed found DISCOVER to have been particularly helpful during their exams, and as a result appeared motivated to continue to employ some of the techniques they learnt. Longer term outcomes are currently being assessed.

### 9.1 Study Limitations

Before drawing our conclusions, certain study limitations need to be acknowledged:

First, the BESST trial was run over two academic years and the trial was delayed for 1 year as schools returned to normal after the COVID‐19 pandemic. All student participants were involved in the first year and we cannot exclude the possibility that some organisational and logistical challenges impacted this cohort more than those recruited in the second year.

Second, the MHST staff we interviewed had limited experience in delivering DISCOVER, indeed the median number of workshops delivered was 1 (range 1–3). It was evident that some team's assessment of the intensity of the training was coloured by the limited number of times they subsequently delivered the intervention.

Third, MHST staff interviews took place in the academic year following their delivery of DISCOVER. Some re‐call problems were evident, though practitioners were clear when this occurred and appropriately cautious.

Fourth, student recruitment was opportunistic and conducted during the school summer holiday. It is likely that the achieved sample differed in certain respects from the trial population. For example, those interviewed possibly had a higher degree of engagement with, or interest in the intervention, or being part of the research.

Fifth, one of the key sampling criteria was to achieve a sub‐sample of male students. Although we were not able to over‐sample male by recruiting one at each site, the male‐to‐female ratio of respondents (27%–73%) matched the trial population (25% male).

## 10 Conclusions

Notwithstanding these limitations, the qualitative findings described above add significant new data which complements the clinical and health economic outcomes measures by BESST. The BESST team concludes that DISCOVER is clinically effective in reducing depressive and anxiety symptoms among adolescents and has potential to be a viable early intervention tool if implemented widely within schools.

The findings of the process evaluation broadly support that position by showing the high level of acceptability which DISCOVER had both for students and the MHSTs who would assume the responsibility for its routine delivery. In some important respects, BESST was an exemplary trial. It recruited to target, and retained and followed up an extremely high proportion of the enroled participants [Brown et al. 2024]. It also successfully trained a relatively new workforce (i.e. EMHPs) to deliver the intervention. Its outcomes were positive, but this does not necessarily mean its performance could not be enhanced.

Should DISCOVER be implemented more widely in routine practice, consideration should be given to reviewing parameters for practitioners to modify scripted material; the time required to cover the breadth of workshop material and whether some material may be delivered digitally to avoid its effectiveness being undermined by student fatigue; and, enhancing support for goal‐attainment.

In relation to goal‐attainment, this may be enhanced by giving more emphasis to the protocol for goal planning, setting and review in the DISCOVER staff training, improved scheduling of the follow‐up calls with clearer explanation to the students of its purpose, and, a flexible goal review method which uses calls or text messaging (according to student preference) from a phone number recognisable to students.

It is well known that the outcomes of trials are difficult to replicate in the real world. But our process evaluation points the way to enhanced real world implementation.

## Ethics Statement

The study was reviewed and approved by King's College London PNM Research Ethics Subcommittee (HR‐20/21–17758).

## Conflicts of Interest

The authors declare no competing interests.

## Participant Consent Statement

All participants provided informed consent before data collection.

## Clinical Trial Registration

The project described in this paper was a process evaluation embedded within the BESST randomised controlled trial, and funded through the trial budget. The BESST trial protocol has been published in full and was registered with ISRCTN (ISRCTN90912799) on May 28, 2020.

## Data Availability

The qualitative data analysed and reported herein have not been shared on any public data repository. Investigators wishing to access the data should contact the corresponding author outlining their analysis plan and research question. All requests will be discussed by the study management group.
